# The Effect of Recombinant Granulocyte Colony-Stimulating Factor on Oral and Periodontal Manifestations in a Patient with Cyclic Neutropenia: A Case Report

**DOI:** 10.1155/2009/654239

**Published:** 2010-02-18

**Authors:** Sergio Matarasso, Vincenzo Daniele, Vincenzo Iorio Siciliano, Michele D. Mignogna, Gianmaria Andreuccetti, Carlo Cafiero

**Affiliations:** Department of Odontostomatological and Maxillo-Facial Science, The School of Medicine and Surgery, Federico II University of Naples, Italy

## Abstract

Cyclic Neutropenia (CN) is characterized by recurrent infections, fever, oral ulcerations, and severe periodontitis as result of the reduced host defences. The previous studies have established the effectiveness of recombinant granulocyte colony-stimulating factor (GCSF) to increase the number and the function of neutrophils in the peripheral blood in this disease. In a 20-year-old Caucasian female with a diagnosis of cyclic neutropenia, oral clinical examination revealed multiple painful ulcerations of the oral mucosa, poor oral hygiene conditions, marginal gingivitis, and moderate periodontitis. The patient received a treatment with G-CSF (Pegfilgrastim, 6 mg/month) in order to improve her immunological status. Once a month nonsurgical periodontal treatment was carefully performed when absolute neutrophil count (ANC) was ≥500/*μ*L. The treatment with G-CSF resulted in a rapid increase of circulating neutrophils that, despite its short duration, leaded to a reduction in infection related events and the resolution of the multiple oral ulcerations. The disappearance of oral pain allowed an efficacy nonsurgical treatment and a normal tooth brushing that determined a reduction of probing depth (PD ≤ 4 mm) and an improvement of the oral hygiene conditions recorded at 6-month follow-up.

## 1. Introduction

Neutrophils play a critical role in the host defence mechanism against bacterial infections [1]. There are 3 general guidelines used to classify theseverity of neutropenia based on the absolute neutrophil count (ANC) measured in cells per microliter of blood:

- mild neutropenia (1000 ≤ ANC < 1500)—minimal risk of infection,
- moderate neutropenia (500 ≤ ANC < 1000)—moderate risk of infection,
- severe neutropenia (ANC < 500)—severe risk of infection.

The above mentioned ranges were developed in Caucasians. In colored population, mild neutropenia is a normal phenomenon, and neutropenia in this population is more properly defined as ANC < 1200. Higher cutoffs may lead to overdiagnosis of neutropenia in the colored population [2]. The clinical consequence of the disease is an increased infective diathesis proportional to the severity of the neutropenia. Several forms of neutropenia (agranulocytosis, familiar benign neutropenia, severe chronic neutropenia) can be found associated with oral manifestations. Severe chronic neutropenia is characterized by a selective decrease in circulating neutrophils and includes a heterogeneous group of haematological diseases divided into three main syndromes: idiopathic neutropenia, congenital forms of neutropenia, and cyclic neutropenia.

The present paper is aimed at evaluating the efficacy of a non surgical periodontal treatment associated with G-CSF therapy in a patient suffering from cyclic neutropenia (CN) associated with oral manifestations and periodontal disease. Written informed consent was obtained by the patient and the authors declare no conflict of interest related to this paper.

## 2. Case Presentation

**Figure 1:**
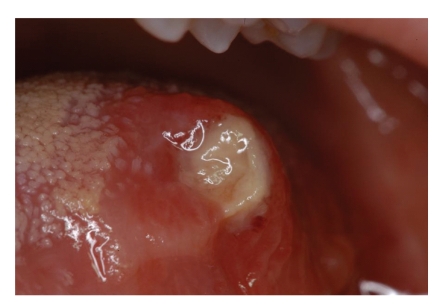
Ulceration of tongue.

**Figure 2:**
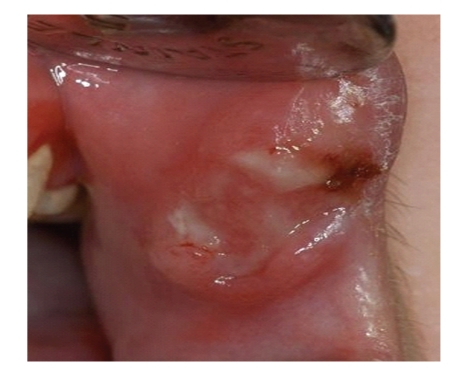
Ulceration of labial commissural.

**Figure 3:**
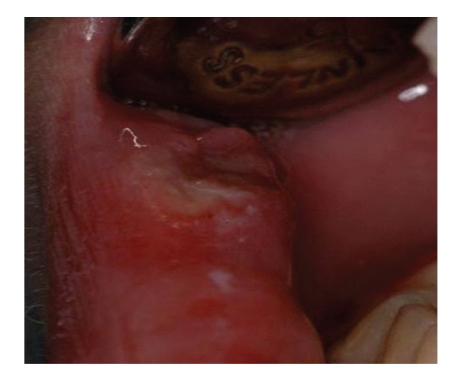
Ulceration of lower lip.

**Figure 4:**
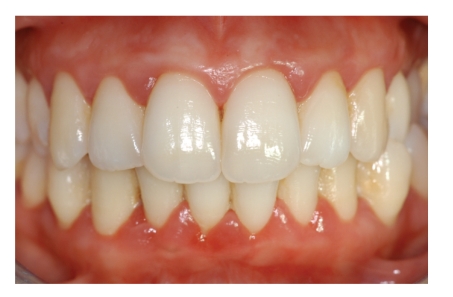
Frontal view of clinical situation at baseline.

**Figure 5:**
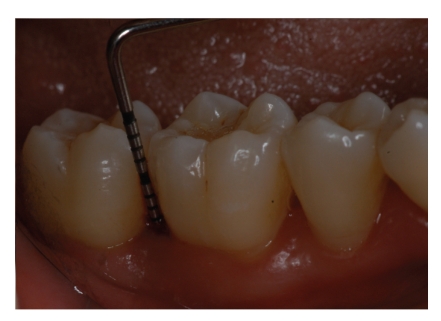
Pathological probing depths were recorded at baseline.

**Figure 6:**
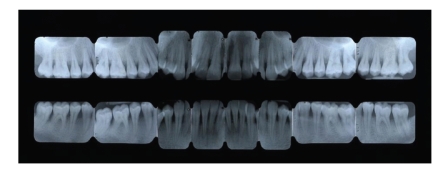
X-ray examination at baseline.

A 20-year-old Caucasian female with diagnosis of CN was referred by the Department of Immunology and Allergology, to the Department of Periodontology of the University of Naples Federico II, Italy. Anamnesis revealed that since the first year of life the patient has been suffering from repeated phases of fever and oral ulcerations, associated with pain and lymphadenopathy. During childhood and adolescence the patient had repeated admissions to the hospital, but CN was diagnosed only in September 2007. The haematological findings (complete laboratory work-up) showed the neutropenic phase recurred approximately every 21 days. The lowest neutrophil count was 0,20 × 10^3^/*μ*L. A light enlargement of submandibular and cervical lymph nodes was revealed by palpation and moreover fever (37,8°C) sinusitis and pharyngitis were recorded during the neutropenic phase. In addition she reported to brush her teeth three times a day but during the neutropenic crisis oral hygiene was hindered by pain due to oropharyngeal ulcerations (Figures 1, 2, and 3). Full Mouth Plaque Score (FMPS) and Full Mouth Bleeding Score (FMBS), recorded on 6 sites per tooth, were 66.7% and 64.8%, respectively (Figure 4). Periodontal examination revealed a generalized increased probing depths (Figure 5). No furcations were involved and no gingival recessions were recorded. Tooth mobility was not present. An X-ray examination revealed bone loss on interproximal aspect of maxillary and mandibular teeth (Figure 6). A diagnosis of moderate periodontitis as manifestation of systemic disease was made.

Since September 2007 up till February 2008 the patient received a systemic treatment with Prednisolone 10 mg/die o.s./7 days (Deltacortene, Bruno Farmaceutici) during the neutropenia phases. The above therapy had a minimum effect on severity of oral ulcerations and produced minimal improvement in ANC. At this regard pain and burning determined poor oral hygiene conditions (FMPS = 41.3% and FMBS = 4.2%).

In March 2008, the patient received a new treatment with GCSF administration consisting of subcutaneous injection of Pegfilgrastim 6 mg (Neupopeg, Dompè Biotec S.p.A., Italy) once a month.

Periodontal treatment including oral hygiene instructions, scaling, root planning, and polishing was meticulously carried out once a month when absolute neutrophil count (ANC) was >1500/*μ*L and for this reason prophylactic antibiotic therapy was not prescribed. During neutropenic cycles the patient was advised to rinse her mouth twice a day with 0.2% chlorhexidine gluconate. A very high level of compliance was recorded. A complete laboratory work-up was performed every week. Therapy with G-CSF initially led to a “peak” of WBC and ANC and subsequently to a normalization for few days (Table 1). No side effects has been reported by the patient during the treatment and no adverse effects were noted when the counts were greater than 40 × 10^3^/*μ*L. A reduction of general and oral manifestations during the neutropenic cycles was constantly recorded. One week after neutropenic phase periodontal re-evaluation was carried out in order to assess the plaque control and the tissutal response to the treatment. 

**Figure 7:**
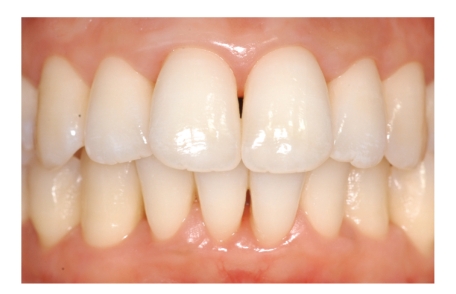
Frontal view of clinical situation after 12-month follow-up period.

**Figure 8:**
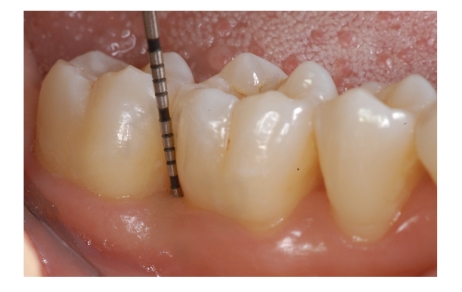
After 12-month follow-up period the periodontal probing show a PD of 3 mm.

A considerable improvement of oral hygiene conditions was observed: FMPS and FMBS remained constantly ≤25% and a period general reduction of probing depth (PD ≤ 4 mm) during the successive 6-month follow-up was recorded (Figures 7 and 8). Microbiological analysis was performed. Microbiological sampling included selection of the deepest periodontal pocket in each quadrant of the dentition based on the probing depth measurements. Sample sites were isolated with cotton rolls and supragingival plaque was carefully removed with curettes and cotton pallets. Subsequently, two paper points were inserted to the depth of the pocket and left in place for 10 seconds.

The paper points were transferred to a vial to be send to the microbiological laboratory in order to perform Polymerase Chain Reaction (PCR, Lab Oral International, The Netherlands). Microbiological analysis revealed no residual pathogenic subgingival microflora but an elevated level of *Fusobacterium nucleatum*.

## 3. Discussion

Cyclic neutropenia (CN) is a chronic rare disorder in the production of the neutrophils, presenting at about 19–21 days intervals. It is clinically characterized by a periodic decrease in the circulating neutrophil numbers determining recurrent infections. 

CN is transmitted by autosomal dominant gene and occurs in infancy but occasionally in adult life [3]. CN is identified as an intrinsic defect of granulocyopoiesis, whose molecular alteration resides in the gene of neutrophil elastase (ELA 2) located on the chromosome 19p13.3. 

In the early maturation stage, the mutation of the gene ELA2 led to an intramedullary apoptosis of neutrophils, that causes regular phases of neutropenia in peripheral blood [4]. The neutropenic phases are typically reported to fall in the range of 19 to 21 days, although recent data indicate that longer periods occur in some patients. The episodes last 5–8 days [5]. The diagnostic criterion of CN is absolute neutrophil count (ANC) less than 0.5 × 10^3^/*μ*L, at least 3 to 5 consecutive days per cycle, for each of three regularly spaced cycles.

As a result of neutropenic phases, haematological findings could reveal increasing in the number of platelets, monocytes, lymphocytes, eosinophils, and reticulocytes [6]. Neutropenia predisposes to respiratory or muco-cutaneous bacterial infection and in some cases was reported in association with otitis media and upper respiratory infection [7]. These manifestations are similar, but less severe, to those commonly reported by patients suffering from agranulocytosis. A general malaise, fever, lymphadenopathy, and oral ulcerations are very common findings during neutropenic periods [8]. Severe periodontitis and oral ulcerations have been reported in the majority of the recorded cases, meanwhile ulcerations may be the unique oral manifestation in about 20% of patients [9–11]. The mucosal lesions can affect any part of the oral mucosa during the neutropenic phases and cause pain and very intense burning that could discourage the patient to maintain a good oral hygiene. Gingivitis is common in CN and severe periodontitis may affect both deciduous and permanent dentitions [12]. In the international literature, severe periodontitis is constantly recorded in subject suffering from CN [13–16], meanwhile this case report is characterized by few periodontal lesions demonstrating that a significant decrease of neutrophil polymorphonucleate leukocytes could be associated with moderate periodontal lesions. Medical management of neutropenias is mainly symptomatic and consists of aggressive antibiotic treatment of febrile patients suspected of having bacterial infections. Other therapies of uncertain efficacy include glucocorticoids, androgenic steroids, immunoglobulins, and plasmapheresis [17–21]. In the last years therapeutic protocols based on the use of recombinant granulocyte colony-stimulating factor (G-CSF) [22–25] that increases for few days the number and the function of neutrophils in the peripheral blood have been proposed by several authors [26–29]. Filgrastim is a granulocyte colony-stimulating factor (G-CSF) analog used to stimulate the proliferation and differentiation of granulocytes. It is produced by recombinant DNA technology and is only slightly different from G-CSF naturally made in humans. Pegfilgrastim is a pegylated form of Filgrastim: pegylation leads to prolongation of its half-life without loss of activity [22, 23]. The recent data demonstrate that Pegfilgrastim, at doses ranging from 6 to 12 mg alone or together with chemotherapy, is capable of efficiently mobilizing peripheral blood stem cells, thus resulting in rapid reconstitution of haematopoiesis. According to data present in the literature, in which no clinically meaningful difference between a single monthly dose of Pegfilgrastim (6 mg) and multiple daily doses of Filgrastim (5 *μ*g/kg/die) was recorded, a single dose of G-CSF (Pegfilgrastim) was monthly administered to the patient [30–33], but there are no organized trials to test or compare Pegfilgrastim versus Filgrastim or other types of G-CSF for treatment of cyclic or chronic neutropenia. 

The monthly therapy with G-CSF caused a rapid increase of circulating neutrophils that, even if of short duration, determined the disappearance of oral ulcerations during the neutropenic phases. These results confirm the outcomes found in the literature, in which the use of G-CSF determined a reduction of oral ulcerations in patient suffering from CN [20]. Moreover the disappearance of ulcerations determined an enhancement of the compliance of the patient who meticulously followed the oral care program. 

At 6-month follow-up nonsurgical treatment of the periodontitis determined a general reduction of probing depth (PD ≤ 4 mm) and a proper tooth brushing leads to an improvement of oral hygiene resulting in an effective plaque control by the patient (FMPS ≤ 25%; FMBS ≤ 25%). At baseline the patient refused to undergo to microbiological analysis and accepted at 6-month follow-up. At 6-month follow-up microbiological analysis revealed no residual pathogenic subgingival microflora but an elevated level of *Fusobacterium nucleatum.*

*Fusobacterium nucleatum *is an anaerobic Gram-negative nonsporeforming oral bacterium found in the normal flora of the mouth, that plays a role in periodontal disease. Because of its opportunistic nature, *Fusobacterium nucleatum *does not affect the environment directly and is not considered a major dental pathogen on its own although it can adhere to a wide range of other organisms, such as *Porphyromonas gingivalis, *and contribute to the development of periodontitis. On the basis of the present microbiological results, a systemic antibiotic therapy was not prescribed to the patient. In conclusion the present results showed that the therapy with recombinant granulocyte colony-stimulating factor (G-CSF) allowed the oral mucosa to heal and influenced the entity of neutropenic cycle. Moreover the periodontal treatment is effective in a patient with CN when compliance is sufficient high to follow the maintenance care program.

## Figures and Tables

**Table 1:** Haematological examinations during G-CSF therapy (March—September 2008).

Date	WBC	NEUTR	LIMPH	MONO
	(10^3^/*μ*L)	(10^3^/*μ*L)	(10^3^/*μ*L)	(10^3^/*μ*L)
17/03/2008	4.87	1.93	2.5	0.19
24/03/2008	3.13	0.86	1.66	0.35
31/03/2008	2.53	0.15	1.44	0.74
05/04/2008	3.35	0.42	1.78	0.86
20/04/2008	2.8	0.04	1.71	0.92

21/04/2008	Subcutaneous injection of G-CSF			

28/04/2008	49.5	43.56	4.16	1.29
06/05/2008	6.44	3.12	2.76	0.32
10/05/2008	4	1.48	2.04	0.36
13/05/2008	3	0.83	1.66	0.39
17/05/2008	2.1	0.05	1.51	0.38
24/05/2008	2.4	0.33	1.29	0.68
29/05/2008	3.43	0.53	1.96	0.66

30/05/2008	Subcutaneous injection of G-CSF			

31/05/2008	4.12	1.03	2.51	0.37
04/06/2008	6.4	0.48	2.49	3.17
08/06/2008	48.93	41.39	4.06	2.15
11/06/2008	21.13	16.48	3.4	0.76
15/06/2008	4.3	2.18	1.78	0.21
18/06/2008	3.4	1.52	1.54	0.22
22/06/2008	4.06	1.49	1.8	0.52
27/06/2008	3.2	0.39	1.77	0.97

28/06/2008	Subcutaneous injection of G-CSF			

29/06/2008	3.67	0.38	1.82	1.01
02/07/2008	21.57	16.22	3.28	1.27
06/07/2008	19.48	13.56	4.36	0.64
09/07/2008	19.41	14.4	3.98	0.58
13/07/2008	4.4	2.17	1.78	0.34
18/07/2008	3.1	1.32	1.64	0.2
22/07/2008	4.16	1.48	1.7	0.51
27/07/2008	3.1	0.34	1.6	0.99

28/07/2008	Subcutaneous injection of G-CSF			

29/07/2008	3.68	0.33	1.8	1.09
02/08/2008	22.47	17.21	3.18	1
06/08/2008	18.47	14.54	4.26	0.54
09/08/2008	18.41	14.43	3.68	0.52
13/08/2008	4.4	2.27	1.88	0.54
17/08/2008	2.8	0.07	1.5	0.35
24/08/2008	2.7	0.35	1.25	0.65
29/08/2008	3.45	0.53	1.88	0.77

30/08/2008	Subcutaneous injection of G-CSF			

31/08/2008	5.12	1.33	2.59	0.39
41/91/2008	6.3	0.48	2.47	3.47
08/09/2008	48.93	41.42	4.06	2.26
11/09/2008	22.43	16.18	3.5	0.77
15/09/2008	4.33	2.28	1.79	0.22

WBC: White blood cell count; Neutr: Neutrophil; Limph: Lymphocytes; Mono: Monocytes.
